# Towards a causative function of histone modifications in regulation of chromatin dynamics and transcription

**DOI:** 10.1186/1756-8935-6-S1-O30

**Published:** 2013-03-18

**Authors:** Philipp Tropberger, Sebastian Pott, Kinga Kamieniarz, Matthieu Caron, Edison T Liu, Raphael Margueron, Robert Schneider

**Affiliations:** 1 Max Planck Institute of lmmunobiology and Epigenetics, 79108 Freiburg, Germany; 2 Cancer Biology and Pharmacology, Genome Institute of Singapore, 138672 Singapore; 3 Institut Curie MR 3215 CNRS, U934INSERM, 26, rue d’Ulm, 75005 Paris, France; 4 Institut de Génétique et de Biologic Moléculaire et Cellulaire, CNRS UMR 7104, INSERM U 964, Université de Strasbourg, 67404 Illkirch, France; 5 Present address: Department of Biology, University of North Carolina at Chapel Hill, NC 27599-3280, USA; 6 Present address: The Jackson Laboratory, 600 Main Street, Bar Harbor, Maine, 04609, USA

## Background

The repertoire of known histone modifications is far from complete. Most attention has been focused on the N-terminal histone tails. However, modifications on the lateral surface of the histone octamer can - potentially - directly regulate nucleosome dynamics. Because of their structurally important position close to the DNA, we can understand what these modifications are actually doing to nucleosome structure and/or dynamics.

## Results

We identified H3K122, a residue at the lateral surface, as a novel acetylation site and use it as a model to study the mechanism of action of lateral surface modifications. By applying a genome-wide approach we find that H3K122ac defines genetic elements associated with transcription and that it co-occurs with active chromatin marks. In line with this H3K122ac is catalyzed by transcriptional co-activators and induced by estrogen receptor signaling. To investigate if H3K122ac not only correlates with active transcription, but has a causative function, we developed an *in vitro* protocol to transcribe chromatin with defined acetylations. We show that chromatin with H3K122ac is transcribed more efficiently than chromatin with unmodified H3, or acetylated at the H3 tail, demonstrating that H3K122ac is capable of stimulating transcription. We show that this is through the relaxation of histone-DNA binding introduced by H3K122ac and the stimulation of nucleosome eviction by H3K122ac. This strongly supports our model that nucleosome function is directly regulated by specific lateral surface modifications.

## Conclusions

Together our findings show that modifications in the nucleosomal core - such as H3K122ac - have a direct role in regulating chromatin function. We demonstrate that one acetylation is sufficient to stimulate transcription by modulating histone-DNA binding. This suggests that transcriptional regulators elicit their effects not only via signaling to histone-tails, but also via direct structural perturbation of nucleosomes by directing acetylation to the lateral octamer surface.

